# Mediterranean diet adherence among women attending mobile breast cancer prevention programme: results of a cross-sectional study in Italy

**DOI:** 10.3389/fpubh.2026.1932170

**Published:** 2026-09-18

**Authors:** Alessandra Maio, Alba Di Leone, Salvatore Di Grande, Gianluca Franceschini, Stefano Magno, Virginia Castagnetta, Riccardo Masetti, Stefania Boccia, Roberta Pastorino

**Affiliations:** 1 Section of Hygiene, University Department of Life Science and Public Health, Università Cattolica del Sacro Cuore, Rome, Italy; 2 Multidisciplinary Breast Center, Department of Woman and Child Health and Public Health, Fondazione Policlinico Universitario A. Gemelli IRCCS, Università Cattolica del Sacro Cuore, Rome, Italy; 3 Department of Woman and Child Health and Public Health, Fondazione Policlinico Universitario A. Gemelli IRCCS, Rome, Italy

**Keywords:** breast cancer, dietary adherence, health promotion, Mediterranean diet, women’s health

## Abstract

**Background:**

The Mediterranean Diet (MedDiet) has been linked to lower breast cancer incidence through anti-inflammatory and antioxidant effects. This study evaluated adherence to the MedDiet among women undergoing mammographic screening provided by mobile units in Italy and explored its association with breast cancer risk factors.

**Materials and methods:**

A cross-sectional study was carried out between January and December 2023 as part of the mobile “Prevention Caravan programme.” Eligible participants included women aged 18 years or older who underwent breast ultrasound or mammography and completed the 14-item “Mediterranean Diet Adherence Screener (MEDAS).” Descriptive analyses and differences across groups defined by known breast cancer risk factors, stratified by MedDiet adherence levels, were performed, and a multivariable logistic regression analysis was conducted to further explore these patterns.

**Results:**

A total of 7,492 women were included, most of whom were ≤45 years of age (69%) and from Central Italy (59%). The mean MEDAS score was 7.0 (SD 1.6), with 62% categorized as having low adherence to the MedDiet. Only 1.21% of participants achieved an excellent level of adherence to the MedDiet. MEDAS score increased with increasing age (*p* = 0.036), women aged 46–74 years and postmenopausal women were also overrepresented among those with excellent adherence (37 and 27% of the excellent-adherence group, respectively; *p* = 0.022 and *p* < 0.001). A significant association emerged with family history of breast conditions (*p* < 0.001), particularly benign lesions, which was associated with lower adherence. These findings were broadly supported by the multivariable analysis.

**Conclusion:**

Mobile programmes like the “Prevention Caravan programme” may help promote healthy lifestyles and improve access to prevention services. Future studies should confirm these results in larger, more diverse populations and explore associations with lesion type and severity.

## 1 Introduction

As a leading cause of morbidity and mortality worldwide, cancer places a substantial burden on public health, profoundly affecting individuals and communities. The World Health Organization (WHO) predicts that by 2050 the number of new cancer cases will exceed 35 million, marking a dramatic 77% increase from the approximately 20 million cases recorded in 2022 (1). Among the various forms of cancer, breast cancer is the most commonly diagnosed malignancy in women and a major health concern. Its high prevalence makes it the leading cause of cancer-related mortality among females (2). Early detection through screening programmes, coupled with effective prevention strategies, is critical—not only to reduce mortality, but also to improve quality of life and reduce healthcare costs associated with treatment and long-term management (3).

Primary prevention, particularly through dietary and lifestyle interventions, plays a crucial role in mitigating breast cancer risk. According to the American Institute for Cancer Research, up to 38% of breast cancer cases may be preventable through a balanced diet and healthy lifestyle choices (4).

Among healthy dietary patterns, the Mediterranean Diet (MedDiet) stands out for its well-documented protective effects against several chronic diseases, including cancer. This dietary model—characterized by consumption of fruits, vegetables, legumes, whole grains, fish, and extra virgin olive oil—has been associated with a significantly reduced risk of breast cancer (5). The MedDiet’s protective effects are primarily attributed to its anti-inflammatory and antioxidant properties, as well as the presence of beneficial nutrients such as monounsaturated and polyunsaturated fatty acids, dietary fibre, and bioactive phytochemicals. These components are known to influence molecular and cellular mechanisms involved in carcinogenesis, thereby reducing the risk of breast cancer development and progression, while also supporting overall health and wellbeing (6, 7).

Although women generally exhibit higher adherence to the MedDiet than men (8), lower adherence is often observed among younger women and those with lower socioeconomic status. This trend—shaped by factors such as education, lifestyle behaviours, and household conditions—combined with declining adherence in certain age groups, highlights the need for targeted public health interventions (9), as also confirmed in the Italian adult population (10).

In Italy, several studies have characterized MedDiet adherence and its socio-demographic determinants in the general adult population, including in women (11), across different regions (12) and over time (13). However, to our knowledge, none of these studies has examined MedDiet adherence among women accessing community-based or mobile breast cancer screening services outside standard organized programmes. Assessing MedDiet adherence in this specific context may help inform targeted nutritional counselling within screening programmes themselves.

In this context, the National Itinerant Programme for Women’s Health Promotion, known as the “Prevention Caravan programme” and organized by Komen Italy, offers free mammographic screenings and health promotion services to women across the country. Since its launch, the initiative has conducted over 918 health promotion days across 17 Italian regions, reaching more than 250,000 women. By facilitating access to screenings and health information, the programme aims not only to improve early detection of breast cancer but also to potentially influence participants’ dietary habits, thereby enhancing their overall health (14).

Within this framework, the primary objective of this study was to assess adherence to the MedDiet, using the “Mediterranean Diet Adherence Screener (MEDAS)” questionnaire (15, 16), among women undergoing mammographic screening as part of the “Prevention Caravan programme.”

Secondary objectives were twofold: (i) to explore how the frequency of MedDiet adherence varied across different categories of known breast cancer risk factors (17); and (ii) to explore the presence of malignant breast lesions among a subset of screened participants who completed a follow-up interview.

## 2 Materials and methods

### 2.1 Endpoints

The primary endpoint was the mean total MEDAS score observed in the study population. The secondary endpoints were: (i) the percentage of the MEDAS score categorized by the different known breast cancer risk factors and (ii) the prevalence of malignant breast lesions in a non-random subgroup of approximately 10% of participants.

### 2.2 Study design, setting, and population

This study adopted a cross-sectional design, with interviews conducted between January and December 2023, as part of a national initiative promoted by Komen Italy, known as the “Prevention Caravan programme.” The programme aims to improve women’s health by offering free prevention activities focused primarily on cancer, especially breast cancer (14).

Mobile units equipped with diagnostic technologies provide mammography, breast ultrasounds, and clinical visits for early diagnosis. These services are delivered by healthcare professionals from the “Fondazione Policlinico Universitario A. Gemelli” and by Komen Italy volunteers, in collaboration with local health units. In addition, the programme offers consultations for women’s health conditions not covered by National Health Service (NHS) screening and educational workshops on physical activity, wellness, and nutrition.

The study population included women ≥18 years who participated in mammographic screening through the “Prevention Caravan programme” in 2023 and who, due to age or other criteria, were not eligible for NHS breast cancer screening programmes. The programme primarily targets women in social or economic hardship—such as those in suburban areas, detention, or shelters—but is open to all women beyond NHS age limits. Awareness was promoted through communication campaigns, and participation facilitated via an online platform.

Women were eligible if they participated in the “Prevention Caravan programme” during the study period, underwent mammographic screening (mammography or breast ultrasound), and completed the MEDAS questionnaire, administered upon arrival at the mobile unit before imaging procedures, thereby reflecting participants’ habitual dietary patterns without influence from educational interventions. Women were excluded if they did not take part in the programme, underwent only non-breast-related screenings or consultations (e.g., for gynecological cancers, thyroid, or dermatological issues), or failed to complete the MEDAS questionnaire.

At a later stage, participants were contacted by phone to collect follow-up information on the outcomes of their breast screening. A non-random subset of 743 women responded based on availability and willingness to participate, providing details on whether any lesions had been detected.

### 2.3 Data collection

Adherence to the MedDiet was evaluated using the 14-item MEDAS questionnaire (Figure 1), a validated tool designed to assess specific dietary behaviours—including the consumption of olive oil, fruits, vegetables, legumes, fish, white meat, and wine—as well as the intake of foods considered inconsistent with the Mediterranean dietary pattern, such as red meat, butter, and sugar-sweetened beverages. The questionnaire was translated into Italian for administration in the present study. Each item is scored as 0 or 1 based on the participant’s response, resulting in a total score ranging from 0 to 14. Higher scores indicate greater adherence: scores greater than or equal to 11 are classified as excellent, scores between 8 and 10 as good, and scores below 8 as low adherence to the MedDiet (15, 16). Of the 7,492 women screened during the study period, all completed the questionnaire, with no missing data recorded for this instrument.

**Figure 1:**
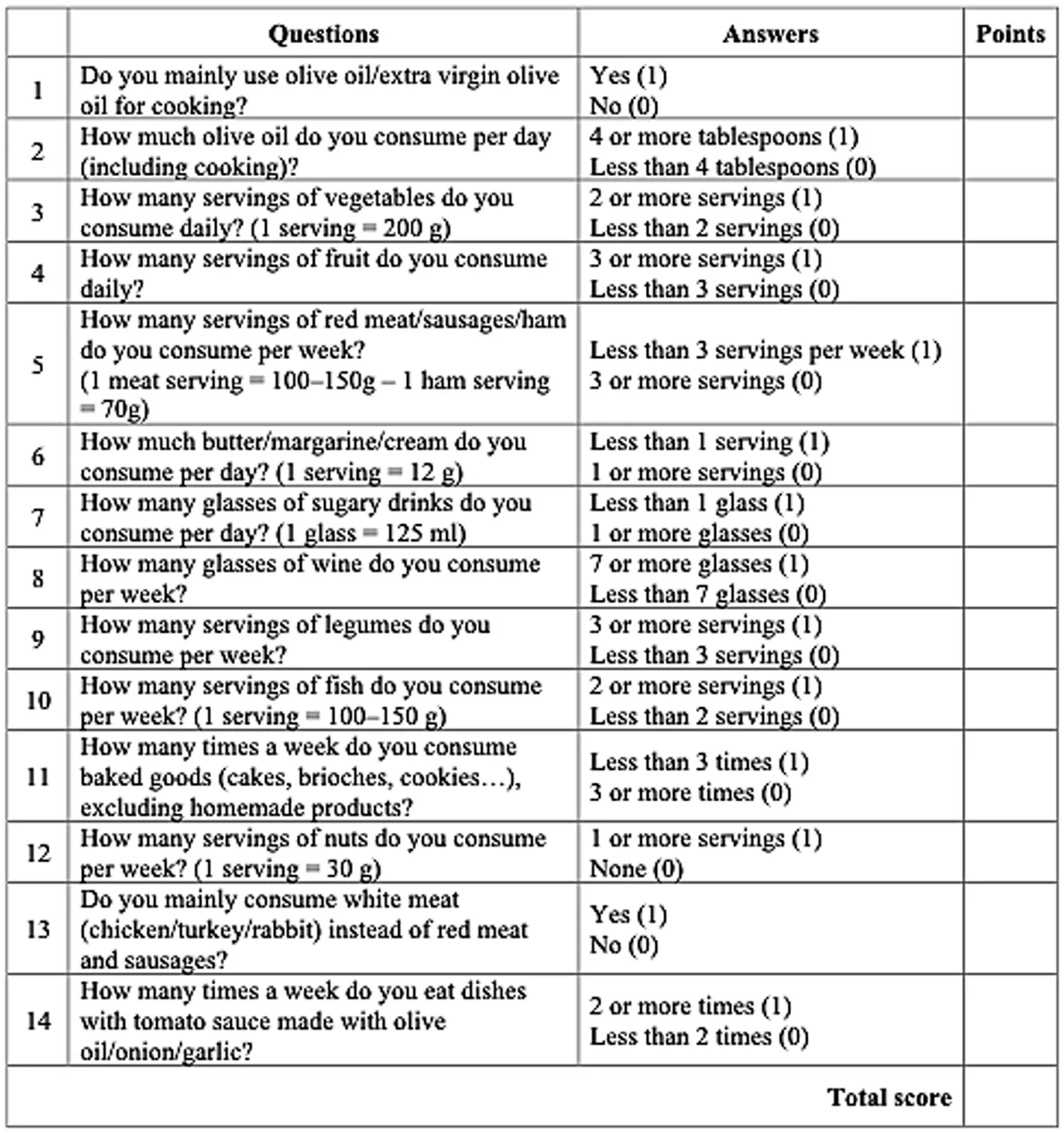
Mediterranean Diet Adherence Screener (MEDAS) questionnaire.

Key breast cancer risk factors were investigated through a structured medical history questionnaire (Supplementary material 1), which collected information on participants’ age, age at menarche, menopausal status, use of hormonal therapies, reproductive history (including number of pregnancies and duration of breastfeeding), and personal or familial history of breast conditions. The categorization criteria applied to each variable, along with their rationale, are reported in Supplementary material 2.

Data on the results of the screening conducted as part of the “Prevention Caravan programme” were available for a non-random subset of 743 out of the 7,492 women included in the study (9.9%), who completed a follow-up telephone interview. While mammographic and ultrasound screening was performed directly within the “Prevention Caravan programme,” women with suspicious findings were referred to local health units for further diagnostic work-up (e.g., biopsy) to confirm or rule out malignancy. As these definitive diagnostic outcomes were not recorded within the programme’s own database, they were collected via self-report during the follow-up telephone interview.

### 2.4 Statistical analysis plan

All analyses were performed using Stata software with a statistical significance level set at *p* < 0.05. Missing values were not imputed. Percentages were calculated among non-missing observations, with missing values reported separately.

The MEDAS score was analyzed both as a continuous variable and as the three-level categorical variable defined above (low, good, and excellent adherence). Breast cancer risk factors were analyzed as continuous or categorical variables, according to the definitions and categorization criteria described in the Supplementary material 2.

Descriptive analyses were initially conducted to characterize the study population. The mean MEDAS score was then calculated for the entire population, and the distribution across the three adherence levels of the categorical MEDAS variable was reported as percentages. Subsequently, the mean values and percentages for the different categories of known breast cancer risk factors were calculated, stratified by adherence level to the MedDiet. Differences across adherence levels were evaluated using the chi-square test or Fisher’s exact test for categorical variables, as appropriate, and the Kruskal-Wallis test for numerical variables, given the non-normal distribution of some of these variables. To further explore factors associated with higher MedDiet adherence, an exploratory multivariable logistic regression model was performed. Because the number of participants with excellent adherence was very small (*n* = 91; 1.21%), good and excellent adherence were combined into a single category and compared with low adherence. The model included age as a continuous variable, geographical area (North, Centre, and South/Islands), pregnancy history (no/yes), and family history of breast disease (none, benign, or malignant). Menopausal status was not included in the multivariable model due to its strong collinearity with age. Southern Italy and Islands were combined because of the small number of participants from the Islands. Adjusted odds ratios (aORs) with 95% confidence intervals (CIs) were estimated.

Also, for the non-random subset of women with available follow-up data, the prevalence of malignant breast lesions was calculated for descriptive purposes only, together with percentages and means for screening results from the “Prevention Caravan programme,” stratified by the categorical MEDAS variable, breast cancer risk factor categories, and other information collected from the medical history questionnaire.

## 3 Results

### 3.1 Characteristics of the study population

The study comprised a total of 7,492 women, of whom 55.02% had undergone breast ultrasound and 44.98% had undergone mammography as part of the “Prevention Caravan programme.” Participants were predominantly from Central Italy (59.15%), followed by Southern Italy (22.83%), Northern Italy (17.75%), and the Islands (0.27%). The mean age of the participants was 41.55 years (±10.08 SD), with the majority (68.85%) aged ≤45 years.

Most participants experienced normal menarche (87.33%), while 10.25% had early menarche and 2.41% had late menarche. Menstrual cycles were regular in the majority of cases (80.90%). Regarding menopausal status, the population was predominantly premenopausal (85.74%), whereas only 11.66% were postmenopausal and 2.60% perimenopausal. The use of hormonal therapies was generally limited: 70.48% of participants had never used them, 19.47% had used them in the past, and 10.04% were undergoing treatment at the time of the study.

In terms of pregnancy history, 56.68% of participants reported having been pregnant. As for breastfeeding, 86.76% of participants had breastfed, with 3.68% currently breastfeeding. Regarding breastfeeding duration, 40.33% breastfed for 6–12 months, 31.56% for more than 12 months, and 28.12% for less than 6 months.

The vast majority of participants had no previous breast lesions (93.46%), while 5.90% had benign lesions and 0.64% had malignant lesions. Regarding familial breast health history, 70.52% of participants reported no family history of breast lesions, 9.35% had a history of benign lesions, and 20.13% had a history of malignant breast lesions. Further detailed information is provided in Table 1.

**Table 1:** Description of the study population.

Variable	Total (*N* = 7,492)
	*N* (%) or Mean ± SD
Mammographic screening	
Breast ultrasound	4,122 (55.02)
Mammography	3,370 (44.98)
*Missing*	*0*
Demographic characteristics	
Geographical area	
Northern Italy	1,305 (17.75)
Central Italy	4,350 (59.15)
Southern Italy	1,679 (22.83)
Islands Italy	20 (0.27)
*Missing*	*138 (1.84)*
Age (continuous)	41.55 ± 10.08
Age (categorical)	
≤45 years	5,001 (68.85)
46–74 years	2,207 (30.38)
≥75 years	56 (0.77)
*Missing*	*228 (3.04)*
Gynecological history	
Menarche age (continuous)	12.35 ± 1.51
Menarche age (categorical)	
Early menarche (≤10 years)	629 (10.25)
Normal menarche (11–15 years)	5,358 (87.33)
Late menarche (≥16 years)	148 (2.41)
*Missing*	*1,357 (18.11)*
Menstrual cycle	
Regular	6,033 (80.90)
Irregular	1,424 (19.10)
*Missing*	*35 (0.46)*
Menopausal status	
Premenopausal (no continuous amenorrhea)	6,065 (85.74)
Perimenopausal (≤12 months of continuous amenorrhea)	184 (2.60)
Postmenopausal (>12 months of continuous amenorrhea)	825 (11.66)
*Missing*	*418 (5.48)*
Hormonal therapies	
Never	5,277 (70.48)
In the past	1,458 (19.47)
Ongoing	752 (10.04)
*Missing*	*5 (0.06)*
Pregnancy	
None	3,180 (43.32)
Yes	4,161 (56.68)
*Missing*	*151 (2.01)*
Breastfeeding	
None	551 (13.24)
Yes	3,610 (86.76)
*Missing*	*0*
Breastfeeding ongoing	
None	3,477 (96.32)
Yes	133 (3.68)
*Missing*	*0*
Breastfeeding time	
<6 months	981 (28.12)
6–12 months	1,407 (40.33)
>12 months	1,101 (31.56)
*Missing*	*121 (1.61)*
Previous personal breast lesion	
None	7,002 (93.46)
Benign	442 (5.90)
Malignant	48 (0.64)
*Missing*	*0*
Previous familial breast lesion	
None	5,270 (70.52)
Benign	699 (9.35)
Malignant	1,504 (20.13)
*Missing*	*19 (0.25)*
Adherence to the MedDiet	
MEDAS score (continuous)	6.99 ± 1.65
MEDAS score (categorical)	
Low adherence (score <8)	4,617 (61.63)
Good adherence (score 8–10)	2,784 (37.16)
Excellent adherence (score ≥11)	91 (1.21)
*Missing*	*0*

MEDAS = Mediterranean diet adherence screener, MedDiet = Mediterranean diet. Column percentages are reported for non-missing values; the proportion of missing data is calculated on the total sample size and reported separately.

### 3.2 Adherence to the MedDiet

The MEDAS score indicated a mean of 6.99 (SD 1.65), reflecting generally low adherence to the MedDiet among the participants. This finding was further supported by categorical analysis, which revealed that 61.63% (*n* = 4,617) of participants exhibited low adherence, 37.16% (*n* = 2,784) demonstrated good adherence, and only 1.21% (*n* = 91) achieved excellent adherence (Table 1). The percentage distribution of responses to each individual MEDAS item is reported in Supplementary material 3.

The distribution of screening type (breast ultrasound vs. mammography) across adherence categories did not differ significantly (*p* = 0.239; Table 2). The geographical distribution, stratified by adherence levels to the MedDiet, differed significantly across adherence categories (*p* = 0.002). Among women with excellent adherence, a higher proportion came from Southern Italy (34.07%).

**Table 2:** Distribution of mammographic screening, geographical area and breast cancer risk factors, stratified by MedDiet adherence level.

Variable	MEDAS score			*p*-value
	Low adherence (score <8) (*N* = 4,617)	Good adherence (score 8–10) (*N* = 2,784)	Excellent adherence (score ≥11) (*N* = 91)	
	*N* (%) or mean ± SD	*N* (%) or mean ± SD	*N* (%) or mean ± SD	
Mammographic screening				0.239
Breast ultrasound	2,571 (55.69)	1,498 (53.81)	53 (58.24)	
Mammography	2,046 (44.31)	1,286 (46.19)	38 (41.76)	
*Missing*	*–*	*–*	*–*	
Geographical area				0.002
Northern Italy	842 (18.69)	450 (16.32)	13 (14.29)	
Central Italy	2,634 (58.47)	1,669 (60.51)	47 (51.65)	
Southern Italy	1,011 (22.44)	637 (23.10)	31 (34.07)	
Islands Italy	18 (0.40)	2 (0.07)	0	
*Missing*	*112 (1.49)*	*26 (0.34)*	*0*	
Breast cancer risk factors				
Age (continuous)	41.29 ± 9.96	41.91 ± 10.13	43.67 ± 13.14	0.036
Age (categorical)				0.022
≤45 years	3,117 (70.00)	1,829 (67.24)	55 (60.44)	
46–74 years	1,303 (29.26)	870 (31.99)	34 (37.36)	
≥75 years	33 (0.74)	21 (0.77)	2 (2.20)	
*Missing*	*164 (2.19)*	*64 (0.85)*	*0*	
Menarche age (continuous)	12.34 ± 1.50	12.37 ± 1.53	12.60 ± 1.35	0.287
Menarche age (categorical)				0.195
Early menarche (≤10 years)	405 (10.73)	219 (9.58)	5 (6.94)	
Normal menarche (11–15 years)	3,289 (87.10)	2,005 (87.67)	64 (88.89)	
Late menarche (≥16 years)	82 (2.17)	63 (2.75)	3 (4.17)	
*Missing*	*841 (11.22)*	*497 (6.63)*	*19 (0.25)*	
Menopausal status				<0.001
Premenopausal (no continuous amenorrhea)	3,776 (86.70)	2,226 (84.64)	63 (70.79)	
Perimenopausal (≤12 months of continuous amenorrhea)	117 (2.69)	65 (2.47)	2 (2.25)	
Postmenopausal (>12 months of continuous amenorrhea)	462 (10.61)	339 (12.89)	24 (26.97)	
*Missing*	*262 (3.50)*	*154 (2.05)*	*2 (0.27)*	
Hormonal therapies				0.643
Never	3,243 (70.29)	1,976 (71.03)	58 (63.74)	
In the past	906 (19.64)	530 (19.05)	22 (24.18)	
Ongoing	465 (10.08)	276 (9.92)	11 (12.09)	
*Missing*	*3 (0.04)*	*2 (0.02)*	*0*	
Pregnancy				0.448
None	1,936 (42.76)	1,206 (44.27)	38 (42.70)	
Yes	2,592 (57.24)	1,518 (55.73)	51 (57.30)	
*Missing*	*89 (1.19)*	*60 (0.80)*	*2 (0.02)*	
Breastfeeding time				0.732
<6 months	608 (28.38)	363 (27.84)	10 (23.26)	
6–12 months	874 (40.80)	516 (39.57)	17 (39.53)	
>12 months	660 (30.81)	425 (32.59)	16 (37.21)	
*Missing*	*80 (1.07)*	*37 (0.49)*	*4 (0.05)*	
Previous personal breast lesion				0.276
None	4,295 (93.03)	2,623 (94.22)	84 (92.31)	
Benign	291 (6.30)	144 (5.17)	7 (7.69)	
Malignant	31 (0.67)	17 (0.61)	0	
*Missing*	*–*	*–*	*–*	
Previous familial breast lesion				<0.001
None	3,215 (69.80)	1,987 (71.58)	68 (74.73)	
Benign	495 (10.75)	202 (7.28)	2 (2.20)	
Malignant	896 (19.45)	587 (21.15)	21 (23.08)	
*Missing*	*11 (0.15)*	*8 (0.11)*	*0*	
Previous breast malignant lesion in a first-degree relative (parents, siblings, children)				0.674
None	263 (69.39)	161 (66.26)	7 (63.64)	
Yes	116 (30.61)	82 (33.74)	4 (36.36)	
*Missing*	*10 (0.13)*	*4 (0.05)*	*0*	

MEDAS = Mediterranean diet adherence screener, MedDiet = Mediterranean diet. Percentages are column percentages, calculated within each adherence category. Column percentages are reported for non-missing values; the proportion of missing data is calculated on the total sample size and reported separately.

Regarding breast cancer risk factors, the mean age increased significantly with higher levels of adherence to the MedDiet (*p* = 0.036). Specifically, the mean age was 41.29 years (SD 9.96) in the low adherence group, 41.91 years (SD 10.13) in the good adherence group, and 43.67 years (SD 13.14) in the excellent adherence group.

Analysis by age group revealed a significant association between age and adherence to the MedDiet (*p* = 0.022). Women aged 46–74 years were disproportionately represented among those with higher adherence: they accounted for 29.26% of women with low adherence, 31.99% of those with good adherence, and 37.36% of those with excellent adherence.

Menarche age did not differ significantly across the adherence groups (*p* = 0.287 for continuous data, *p* = 0.195 for categorical data). In contrast, menopausal status was significantly associated with levels of adherence to the MedDiet (*p* < 0.001): postmenopausal women were disproportionately represented among those with higher adherence, accounting for 10.61% of women with low adherence, 12.89% of those with good adherence, and 26.97% of those with excellent adherence.

There was no significant difference between adherence and the use of hormonal therapies (*p* = 0.643), pregnancy history (*p* = 0.448), or breastfeeding duration (*p* = 0.732).

Regarding the history of breast diseases, no significant difference was found between personal history and adherence to the MedDiet, whereas a significant difference was observed with family history (*p* < 0.001). Specifically, the proportion of women reporting a family history of benign breast lesions decreased with increasing MedDiet adherence: 10.75% in the low-adherence group, 7.28% in the good-adherence group, and 2.20% in the excellent-adherence group. When considering the presence of a previous malignant breast lesion in a first-degree relative (parents, siblings, children), the difference between the various MedDiet adherence groups was not significant (*p* = 0.674).

A multivariable logistic regression analysis showed results broadly consistent with the patterns observed in the descriptive analyses (Table 3). Increasing age was associated with a higher likelihood of adherence to the MedDiet (aOR 1.01, 95% CI 1.00–1.01, *p* = 0.002). Compared to Northern Italy, living in Central Italy (aOR 1.17, 95% CI 1.03–1.34, *p* = 0.018) and in Southern Italy and Islands (aOR 1.19, 95% CI 1.02–1.38, *p* = 0.029) was associated with higher adherence. A positive pregnancy history was associated with a lower likelihood of adherence (aOR 0.87, 95% CI 0.77–0.97, *p* = 0.012), as was a family history of benign breast disease (aOR 0.71, 95% CI 0.59–0.85, *p* < 0.001), while no significant difference was observed for a family history of malignant breast disease (*p* = 0.393).

**Table 3:** Multivariable logistic regression analysis of factors associated with adherence to the MedDiet (good/excellent adherence to the Mediterranean diet vs. low adherence).

Variable	aOR	95% CI	*p*-value
Age (per year)	1.01	1.00–1.01	0.002
Geographical area			
North (ref.)	1.00	–	–
Centre	1.17	1.03–1.34	0.018
South/Islands	1.19	1.02–1.38	0.029
Pregnancy history			
None (ref.)	1.00	–	–
Yes	0.87	0.77–0.97	0.012
Family history of breast disease			
None (ref.)	1.00	–	–
Benign	0.71	0.59–0.85	<0.001
Malignant	1.05	0.93–1.19	0.393

### 3.3 Screening results from the “Prevention Caravan programme”

The prevalence of malignant breast lesions diagnosed through screening was evaluated in a non-random subgroup of 743 women from the original cohort of 7,492 participants (9.9%), representing those who responded to follow-up contact. The characteristics of this subgroup are presented descriptively alongside those of non-responders in Supplementary Table S4A. As responders and non-responders were not randomly allocated, the findings presented in this section should be interpreted with appropriate caution.

The results showed that 1.08% (*n* = 8) of these women had malignant lesions (95% CI: 0.0054–0.0214).

Low adherence to the MedDiet, as indicated by the MEDAS score, was predominant in both groups: 7 of 8 women with malignant lesions and 605 of 735 women without malignant lesions. Good adherence was observed in 1 of 8 women with malignant lesions and 130 of 735 women without, while no participants in either group exhibited excellent adherence.

Further detailed information can be found in Supplementary material 4.

## 4 Discussion

The aim of this study was to assess adherence to the MedDiet among women who participated in the national free mammography screening programme known as the “Prevention Caravan programme.” This population primarily includes women who, due to age or socio-economic conditions, are not eligible for organized screening programmes offered by the NHS and may therefore be more vulnerable or less likely to be reached by conventional prevention initiatives. Assessing their dietary behaviours represents a crucial first step in identifying specific needs and developing targeted, integrated health promotion strategies. In this context, incorporating food insecurity screening tools may help identify individuals at greater risk and support the implementation of tailored interventions to improve participation in breast cancer screening (18, 19).

The results showed generally a low adherence to the MedDiet, with only 1.2% of participants achieving an “excellent” score. Similar findings have been reported in studies conducted in Italy and other Mediterranean countries, documenting a steady decline in adherence to this dietary pattern, particularly among younger adults and socioeconomically disadvantaged groups (13, 20, 21). This pattern is corroborated by more recent Italian surveys, which similarly report suboptimal adherence to the Mediterranean dietary pattern and a parallel rise in the consumption of ultra-processed foods over the past 15 years, reinforcing the consistency of this trend across independent samples and study periods (22, 23). Factors such as education level, income, and the perception of the diet as healthy and sustainable are positively associated with adherence (11, 20). In particular, findings from the Moli-sani cohort showed a marked reduction in adherence among lower-income groups following the economic crisis, highlighting the influence of social inequalities on dietary choices (24).

Despite this evidence, women from Southern Italy were more frequently represented in the group with excellent adherence compared to those from Central and Northern Italy. This geographical pattern likely reflects the persistence of traditional eating habits in Southern areas, where access to typical Mediterranean foods remains more widespread (10). However, this cultural advantage does not necessarily translate into better health outcomes: Southern Italy continues to report higher rates of obesity, diabetes, and sedentary behaviour (25). These contradictions may be due to a progressive erosion of the MedDiet, especially among younger generations, and the growing consumption of ultra-processed foods, sugary drinks, and low-cost industrial products (26). Additionally, adverse social conditions—such as low education levels, family-related stress, and limited access to health resources—contribute to poorer dietary quality and physical inactivity, even in regions where the MedDiet remains culturally relevant (10, 11).

Adherence to the MedDiet was significantly higher among older women and those who were postmenopausal. This result is consistent with national studies showing that adherence increases progressively with age and is highest among adults aged ≥75 years.

A significant association was found between adherence to the MedDiet and family history of breast conditions, driven primarily by benign breast disease. Specifically, women reporting such a family history did not report healthier eating behaviours. This finding is supported by existing literature showing that, although individuals with a family history of breast disease may perceive a higher personal risk, they do not consistently adopt healthier lifestyle behaviours (27, 28). The only preventive behaviour more frequently observed in this group is greater participation in screening programmes (29).

Similarly, no associations were observed between dietary adherence and hormonal or reproductive factors such as age at menarche, pregnancy history, breastfeeding, or hormone therapy use. Some studies have suggested a link between adherence to the MedDiet and later menarche, although the evidence remains limited and inconclusive (30). While adherence tends to be lower during pregnancy (31), it generally improves in the postmenopausal period, likely reflecting increased health consciousness and more consistent lifestyle habits (32).

Overall, the multivariable analysis supported the findings observed in the primary analyses for age, geographical area, and family history of benign breast disease, while also suggesting lower MedDiet adherence among women with a positive pregnancy history. Interestingly, pregnancy history was not associated with MedDiet adherence in the bivariate analysis but emerged as an independent predictor in the multivariable model. This discrepancy is likely explained by confounding by age, which was strongly associated with adherence and is inherently correlated with pregnancy history; adjustment for age in the multivariable model may have unmasked an independent inverse association between pregnancy history and MedDiet adherence, consistent with evidence that maternal age is itself an independent determinant of dietary adherence during the reproductive years (33).

The prevalence of malignant breast lesions in the limited follow-up sample was small, preventing any reliable conclusions about the association between MedDiet and breast cancer outcomes. Adherence to the MedDiet was low in both women with and without malignant lesions, although the limited number of malignant lesions precludes any meaningful interpretation of this comparison.

These findings carry specific implications for public health practice within mobile screening programmes. Rather than being limited to general encouragement of healthy eating, MEDAS score results could be used to deliver real-time, individualised dietary feedback at the point of screening, allowing women identified with low adherence to receive immediate, targeted counselling. mHealth tools have shown promise in supporting sustained engagement with dietary behaviour change and could be leveraged to maintain contact with participants and support follow-up beyond the single screening encounter (34, 35). Cancer screening visits have similarly been identified as a “teachable moment” during which women are particularly receptive to lifestyle advice (36), reinforcing the potential value of embedding dietary counselling within the “Prevention Caravan programme” itself. Precedents for this model exist: the ActWELL trial embedded a volunteer-delivered lifestyle programme within the Scottish NHS breast screening programme, using the screening appointment as the point of recruitment for a structured follow-up intervention on weight, physical activity, and alcohol consumption (37, 38). A systematic review and meta-analysis of diet and physical activity interventions initiated through breast and colorectal cancer screening reported modest but significant improvements in body weight, BMI, physical activity, and fruit and vegetable intake, although evidence quality was generally low (39). Notably, none of the identified studies specifically addressed mobile or community-based screening programmes, and referral was typically delivered by dedicated coaches rather than screening staff themselves, indicating that further work is needed to adapt and test this model within initiatives such as the “Prevention Caravan programme.”

This study has several limitations that must be considered when interpreting the findings. The cross-sectional design limits the ability to establish causal relationships between adherence to the MedDiet and breast cancer risk factors. Although multivariable analyses were performed, the study design does not allow the exclusion of residual confounding. Additionally, the study population consisted primarily of women who voluntarily participated in a free screening programme, potentially introducing selection bias, as these women may differ from the general population in terms of socioeconomic status, health awareness, or access to healthcare. Although the “Prevention Caravan programme” is specifically designed to improve access to screening among women potentially underserved by institutional pathways, no individual-level socioeconomic variables were collected. Likewise, data on lifestyle-related confounders such as body mass index and smoking status were not available. The absence of these variables may result in residual confounding in the multivariable analysis, and this should be taken into account when interpreting the observed associations. The lack of detailed data on some relevant variables—such as the duration of hormone therapy—may affect the accuracy of risk factor assessment. The use of self-reported data for dietary adherence and medical history could introduce recall bias. Objective dietary assessments and clinical verification of risk factors would enhance the reliability of the findings. The absence of longitudinal follow-up further limits our understanding of how dietary patterns evolve over time and their potential impact on long-term health outcomes. Finally, the small number of malignant lesions identified in the follow-up sample and the non-random nature of the subgroup require that these findings be interpreted as descriptive only and not representative of the entire study cohort.

## 5 Conclusion

This study highlights a generally low adherence to the MedDiet among women participating in the “Prevention Caravan programme” in Italy. These findings emphasize the need to incorporate targeted nutritional education into public health strategies aimed at Italian women, particularly younger age groups. Leveraging the reach and accessibility of mobile screening programmes such as the “Prevention Caravan programme” may offer an innovative public health opportunity to promote healthy lifestyles and improve access to prevention services among populations less likely to be reached by organized screening programmes, for example by using MEDAS score results to deliver real-time dietary counselling, integrating mHealth tools to sustain participant engagement and follow-up, and establishing formal referral pathways linking screening initiatives with primary care nutrition services.

Considering the limitations of this study, future research should aim to confirm these findings in larger and more diverse samples, in order to assess whether they accurately reflect dietary habits across different regions of Italy, and should incorporate broader socioeconomic and lifestyle data (e.g., education, income, BMI, smoking status) to allow more comprehensive adjustment for potential confounding. Further investigation is also needed to explore whether dietary adherence varies not only with a greater number of malignant lesions, but also in relation to the specific type of lesion detected. Future studies could also build on these findings by examining adherence to individual MEDAS items, which may help identify specific dietary components driving low adherence and inform targeted nutritional counselling within mobile screening programmes.

## Data Availability

The raw data supporting the conclusions of this article will be made available by the authors, without undue reservation.
