# Supplementary material for: Mediterranean diet adherence among women attending mobile breast cancer prevention programme: results of a cross-sectional study in Italy
Source: Front Public Health. 2026 Sep 18;14:1932170. doi: 10.3389/fpubh.2026.1932170 (PMC13630691; doi:10.3389/fpubh.2026.1932170)
Supplement: Supplementary file 1 [file Table_1.docx]

**Supplementary Material 1. Medical history questionnaire**

Date of last mammogram: ____ / ____ / ________

Date of last breast ultrasound: ____ / ____ / ________

Breastfeeding:	□ Yes □ No

Currently breastfeeding:	□ Yes □ No

For how long have you breastfed?	__________

Family history of breast cancer:	□ Yes □ No

Degree of kinship: ____________________

Age at diagnosis: ______

Age at menarche: __________	Last Menstrual Period/Menopause: _____________

Pregnancies: __________________

Hormonal therapy/HRT:	□ Ongoing □ Never taken □ In the past

Previous breast disease:

□ Malignant	□ Right	□ Left

□ Benign	□ Right	□ Left

Age at diagnosis: __________

Previous breast surgery:

____________________________________________________

Other procedures (if any):

____________________________________________________

Have you ever participated in Komen Italia screenings?	□ Yes □ No
