# Supplementary material for: Mediterranean diet adherence among women attending mobile breast cancer prevention programme: results of a cross-sectional study in Italy
Source: Front Public Health. 2026 Sep 18;14:1932170. doi: 10.3389/fpubh.2026.1932170 (PMC13630691; doi:10.3389/fpubh.2026.1932170)
Supplement: Supplementary file 2 [file Table_2.docx]

**Supplementary Material 2**

**Table S2. Definitions and categorization criteria of the study covariates**

| **Variable** | **Categorization** | **Rationale** |
| --- | --- | --- |
| Age | - ≤45 years - 46–74 years - ≥75 years | The lower cut-point (45 years) corresponds to the age at which the American Cancer Society recommends that women at average risk begin regular annual mammographic screening. The upper cut-point (75 years) corresponds to the age beyond which the continuation of screening mammography becomes a matter of individualized clinical judgement, rather than a uniform recommendation, across current national and international guidelines. This three-category classification also reproduces the age grouping adopted in a large national cohort study comparing tumor histopathology, stage at presentation, and treatment at the extremes of age [1-3]. |
| Menarche age | - Early menarche (≤10 years) - Normal menarche (11–15 years) - Late menarche (≥16 years) | Based on the 5^th^ and 95^th^ percentile cut-points for age at menarche identified in a large prospective cohort study, which used the same three categories to identify women at increased long-term metabolic risk [4]. |
| Menopausal status | - Premenopausal (no continuous amenorrhea) - Perimenopausal (≤12 months of continuous amenorrhea) - Postmenopausal (>12 months of continuous amenorrhea) | Based on WHO/STRAW+10 clinical criteria, whereby natural menopause is defined as occurring after 12 consecutive months of amenorrhea, and perimenopause as the transitional phase ending at that 12-month mark [5]. |
| Hormonal therapies | - Never - In the past - Ongoing | Categorization reflects standard exposure-status classification (never/past/ongoing use) commonly applied to treatment exposure variables. |
| Pregnancies | - None - Yes | Binary classification based on presence or absence of at least one pregnancy. |
| Breastfeeding time | - 0 months - <6 months - 6–12 months - >12 months | Reflects WHO public health recommendations on breastfeeding practice (exclusive breastfeeding for the first 6 months, continued breastfeeding up to 2 years or beyond), providing clinically and publicly meaningful cut-points [6]. |
| Previous personal breast lesion | - None - Benign (e.g., fibroadenomas, breast cysts, fibrocystic breast disease) - Malignant (breast cancer) | Categorization based on clinical nature of the lesion (absent/benign/malignant). |
| Previous familial breast lesion | - None - Benign (e.g., fibroadenomas, breast cysts, fibrocystic breast disease) - Malignant (breast cancer) | Categorization based on clinical nature of the lesion (absent/benign/malignant). |
| Previous malignant lesion in a first-degree relative | - None - Yes | Binary classification based on presence or absence of malignant breast lesion in a first-degree relative, a well-established breast cancer risk factor [7]. |
| Screening result (“Prevention Caravan programme”) | - No malignant lesion - Malignant lesion | Binary classification based on the outcome of the screening examination (absence/presence of malignant lesion). |

**References**

1. Lee CS, Lewin A, Reig B et al. Women 75 Years Old or Older: To Screen or Not to Screen? Radiographics 2023;43, DOI: 10.1148/RG.220166.
2. Plichta JK, Thomas SM, Vernon R et al. Breast cancer tumor histopathology, stage at presentation, and treatment in the extremes of age. Breast Cancer Res Treat 2020;180:227–35.
3. Oeffinger KC, Fontham ETH, Etzioni R et al. Breast cancer screening for women at average risk: 2015 Guideline update from the American cancer society. JAMA - Journal of the American Medical Association 2015;314:1599–614.
4. Glueck CJ, Morrison JA, Wang P et al. Early and late menarche are associated with oligomenorrhea and predict metabolic syndrome 26 years later. Metabolism 2013;62:1597–606.
5. Ambikairajah A, Walsh E, Cherbuin N. A review of menopause nomenclature. Reprod Health 2022;19:1–15, DOI: 10.1186/s12978-022-01336-7.
6. World Health Organization. Infant and young child feeding [fact sheet]. Geneva: WHO. Available from: <https://www.who.int/news-room/fact-sheets/detail/infant-and-young-child-feeding>.
7. Ahern TP, Sprague BL, Bissell MCS et al. Family history of breast cancer, breast density, and breast cancer risk in a U.S. breast cancer screening population. Cancer Epidemiology Biomarkers and Prevention 2017;26:938–44.
