# Supplementary material for: Mediterranean diet adherence among women attending mobile breast cancer prevention programme: results of a cross-sectional study in Italy
Source: Front Public Health. 2026 Sep 18;14:1932170. doi: 10.3389/fpubh.2026.1932170 (PMC13630691; doi:10.3389/fpubh.2026.1932170)
Supplement: Supplementary file 3 [file Table_3.docx]

**Supplementary Material 3**

**Table S3. Item-level response distribution for the 14-item MEDAS questionnaire (N = 7,492)**

|  | **Total**  **(N = 7,492)** |
| --- | --- |
| **MEDAS item and response category** | **N (%)** |
| **1. Do you mainly use olive oil/extra virgin olive oil for cooking?** |  |
| Yes (1 point) | 6,693 (89.34) |
| No (0 points) | 799 (10.66) |
| **2. How much olive oil do you consume per day (including cooking)?** |  |
| 4 or more tablespoons (1 point) | 3,324 (44.37) |
| Less than 4 tablespoons (0 points) | 4,168 (55.63) |
| **3. How many servings of vegetables do you consume daily? (1 serving = 200 g)** |  |
| 2 or more servings (1 point) | 20 (0.27) |
| Less than 2 servings (0 points) | 7,472 (99.73) |
| **4. How many servings of fruit do you consume daily?** |  |
| 3 or more servings (1 point) | 731 (9.76) |
| Less than 3 servings (0 points) | 6,761 (90.24) |
| **5. How many servings of red meat/sausages/ham do you consume per week? (1 meat serving = 100–150g – 1 ham serving = 70g)** |  |
| Less than 3 servings per week (1 point) | 7,105 (94.83) |
| 3 or more servings (0 points) | 387 (5.17) |
| **6. How much butter/margarine/cream do you consume per day? (1 serving = 12 g)** |  |
| Less than 1 serving (1 point) | 6,219 (83.01) |
| 1 or more servings (0 points) | 1,273 (16.99) |
| **7. How many glasses of sugary drinks do you consume per day? (1 glass = 125 ml)** |  |
| Less than 1 glass (1 point) | 5,347 (71.37) |
| 1 or more glasses (0 points) | 2,145 (28.63) |
| **8. How many glasses of wine do you consume per week?** |  |
| 7 or more glasses (1 point) | 557 (7.43) |
| Less than 7 glasses (0 points) | 6,935 (92.57) |
| **9. How many servings of legumes do you consume per week?** |  |
| 3 or more servings (1 point) | 1,189 (15.87) |
| Less than 3 servings (0 points) | 6,303 (84.13) |
| **10. How many servings of fish do you consume per week? (1 serving = 100–150 g)** |  |
| 2 or more servings (1 point) | 2,846 (37.99) |
| Less than 2 servings (0 points) | 4,646 (62.01) |
| **11. How many times a week do you consume baked goods (cakes, brioches, cookies…), excluding homemade products?** |  |
| Less than 3 times (1 point) | 3,628 (48.42) |
| 3 or more times (0 points) | 3,864 (51.58) |
| **12. How many servings of nuts do you consume per week? (1 serving = 30 g)** |  |
| 1 or more servings (1 point) | 5,161 (68.89) |
| None (0 points) | 2,331 (31.11) |
| **13. Do you mainly consume white meat (chicken/turkey/rabbit) instead of red meat and sausages?** |  |
| Yes (1 point) | 5,039 (67.26) |
| No (0 points) | 2,453 (32.74) |
| **14. How many times a week do you eat dishes with tomato sauce made with olive oil/onion/garlic?** |  |
| 2 or more times (1 point) | 4,507 (60.16) |
| Less than 2 times (0 points) | 2,985 (39.84) |

**Abbreviations:**

MEDAS = Mediterranean Diet Adherence Screener.
