# Supplementary material for: Mediterranean diet adherence among women attending mobile breast cancer prevention programme: results of a cross-sectional study in Italy
Source: Front Public Health. 2026 Sep 18;14:1932170. doi: 10.3389/fpubh.2026.1932170 (PMC13630691; doi:10.3389/fpubh.2026.1932170)
Supplement: Supplementary file 4 [file Table_4.docx]

**Supplementary Material 4**

**Table S4 A. Characteristics of responders and non-responders to the follow-up telephone interview**

|  | **Non-responder**  **(N = 6,749)** | **Responder**  **(N = 743)** |
| --- | --- | --- |
|  | **N (%) or**  **Mean ± SD** | **N (%) or**  **Mean ± SD** |
| **Mammographic screening** |  |  |
| Breast ultrasound | 3,807 (56.41) | 315 (42.40) |
| Mammography | 2,942 (43.59) | 428 (57.60) |
| *Missing* | *0* | *0* |
| **Demographic characteristics** |  |  |
| Geographical area |  |  |
| Northern Italy | 1,234 (18.28) | 71 (11.74) |
| Central Italy | 3,923 (58.13) | 427 (70.58) |
| Southern Italy | 1,592 (23.59) | 87 (14.38) |
| Islands Italy | 0 | 20 (3.31) |
| *Missing* | *0* | *138 (1.84)* |
| Age (continuous) | 41.58 ± 10.21 | 41.19 ± 8.64 |
| Age (categorical) |  |  |
| ≤45 years | 4,542 (68.65) | 459 (70.83) |
| 46-74 years | 2,020 (30.53) | 187 (28.86) |
| ≥75 years | 54 (0.82) | 2 (0.31) |
| *Missing* | *133 (1.78)* | *95 (1.27)* |
| **Gynecological history** |  |  |
| Menarche age (continuous) | 12.36 ± 1.52 | 12.33 ± 1.47 |
| Menarche age (categorical) |  |  |
| Early menarche (≤10 years) | 548 (10.15) | 81 (11.02) |
| Normal menarche (11-15 years) | 4,714 (87.30) | 644 (87.62) |
| Late menarche (≥16 years) | 138 (2.56) | 10 (1.36) |
| *Missing* | *1,349 (18.01)* | *8 (0.11)* |
| Menstrual cycle |  |  |
| Regular | 5,479 (81.56) | 554 (74.97) |
| Irregular | 1,239 (18.44) | 185 (25.03) |
| *Missing* | *31 (0.41)* | *4 (0.05)* |
| Menopausal status |  |  |
| Premenopausal (no continuous amenorrhea) | 5,442 (84.36) | 623 (100.00) |
| Perimenopausal (≤12 months of continuous amenorrhea) | 184 (2.85) | 0 |
| Postmenopausal (>12 months of continuous amenorrhea) | 825 (12.79) | 0 |
| *Missing* | *298 (3.98)* | *120 (1.60)* |
| Hormonal therapies |  |  |
| Never | 4,863 (72.07) | 414 (56.02) |
| In the past | 1,226 (18.17) | 232 (31.39) |
| Ongoing | 659 (9.77) | 93 (12.58) |
| *Missing* | *1 (0.01)* | *4 (0.05)* |
| Pregnancy |  |  |
| None | 2,872 (43.50) | 308 (41.68) |
| Yes | 3,730 (56.50) | 431 (58.32) |
| *Missing* | *147 (1.96)* | *4 (0.05)* |
| Breastfeeding |  |  |
| None | 3,521 (52.17) | 361 (48.59) |
| Yes | 3,228 (47.83) | 382 (51.41) |
| *Missing* | *0* | *0* |
| Breastfeeding ongoing |  |  |
| None | 6,616 (98.03) | 743 (100.00) |
| Yes | 133 (1.97) | 0 |
| *Missing* | *0* | *0* |
| Breastfeeding time |  |  |
| <6 months | 878 (28.22) | 103 (27.25) |
| 6-12 months | 1,263 (40.60) | 144 (38.10) |
| >12 months | 970 (31.18) | 131 (34.66) |
| *Missing* | *117 (1.56)* | *4 (0.05)* |
| Previous personal breast lesion |  |  |
| None | 6,334 (93.85) | 668 (89.91) |
| Benign | 383 (5.67) | 59 (7.94) |
| Malignant | 32 (0.47) | 16 (2.15) |
| *Missing* | *0* | *0* |
| Previous familial breast lesion |  |  |
| None | 4,787 (71.37) | 460 (61.91) |
| Benign | 416 (6.20) | 283 (38.09) |
| Malignant | 1,504 (22.42) | 0 |
| *Missing* | *42 (0.56)* | *0* |
| **Adherence to the MedDiet** |  |  |
| MEDAS score (continuous) | 7.07 ± 1.67 | 6.28 ± 1.33 |
| MEDAS score (categorical) |  |  |
| Low adherence (score <8) | 4,005 (59.34) | 612 (82.37) |
| Good adherence (score 8-10) | 2,653 (39.31) | 131 (17.63) |
| Excellent adherence (score ≥11) | 91 (1.35) | 0 |
| *Missing* | *0* | *0* |

**Notes:**

Column percentages are reported for non-missing values; the proportion of missing data is calculated on the total sample size and reported separately.

**Table S4 B. Distribution of mammographic screening result from the “Prevention Caravan programme” across geographical area and breast cancer risk factor categories**

|  | **Screening result from the**  **“*Prevention Caravan programme*”** | |
| --- | --- | --- |
|  | **No malignant lesion**  **(N = 735)** | **Malignant lesion**  **(N = 8)** |
|  | **N (%) or**  **Mean ± SD** | **N (%) or**  **Mean ± SD** |
| **Mammographic screening** |  |  |
| Breast ultrasound | 315 (42.86) | 0 |
| Mammography | 420 (57.14) | 8 (100.00) |
| *Missing* | *0* | *0* |
| **MEDAS score** |  |  |
| Low adherence (score <8) | 605 (82.31) | 7 (87.50) |
| Good adherence (score 8-10) | 130 (17.69) | 1 (12.50) |
| Excellent adherence (score ≥11) | 0 | 0 |
| *Missing* | *0* | *0* |
| **Demographic characteristics** |  |  |
| Geographical area |  |  |
| Northern Italy | 71 (11.87) | 0 |
| Central Italy | 423 (70.74) | 4 (57.14) |
| Southern Italy | 86 (14.38) | 1 (14.29) |
| Islands Italy | 18 (3.01) | 2 (28.57) |
| *Missing* | *137 (18.44)* | *1 (0.13)* |
| Age (continuous) | 41.10 ± 8.65 | 47.75 ± 4.33 |
| Age (categorical) |  |  |
| ≤45 years | 457 (71.41) | 2 (25.00) |
| 46-74 years | 181 (28.28) | 6 (75.00) |
| ≥75 years | 2 (0.31) | 0 |
| *Missing* | *95 (12.79)* | *0* |
| **Gynecological history** |  |  |
| Menarche age (continuous) | 12.32 ± 1.46 | 13 ± 2 |
| Menarche age (categorical) |  |  |
| Early menarche (≤10 years) | 79 (10.87) | 2 (25.00) |
| Normal menarche (11-15 years) | 638 (87.76) | 6 (75.00) |
| Late menarche (≥16 years) | 10 (1.38) | 0 |
| *Missing* | *8 (1.08)* | *0* |
| Menstrual cycle |  |  |
| Regular | 550 (75.24) | 4 (50.00) |
| Irregular | 181 (24.76) | 4 (50.00) |
| *Missing* | *4 (0.54)* | *0* |
| Hormonal therapies |  |  |
| Never | 410 (56.09) | 4 (50.00) |
| In the past | 231 (31.60) | 1 (12.50) |
| Ongoing | 90 (12.31) | 3 (37.50) |
| *Missing* | *4 (0.54)* | *0* |
| Pregnancy |  |  |
| None | 314 (42.95) | 1 (12.50) |
| Yes | 417 (57.05) | 7 (87.50) |
| *Missing* | *4 (0.54)* | *0* |
| Breastfeeding |  |  |
| None | 53 (12.71) | 0 |
| Yes | 364 (87.29) | 7 (100.00) |
| *Missing* | *0* | *0* |
| Breastfeeding time |  |  |
| <6 months | 104 (28.57) | 1 (14.29) |
| 6-12 months | 134 (36.81) | 4 (57.14) |
| >12 months | 126 (34.62) | 2 (28.57) |
| *Missing* | *0* | *0* |
| Previous personal breast lesion |  |  |
| None | 665 (90.48) | 3 (37.50) |
| Benign | 58 (7.89) | 1 (12.50) |
| Malignant | 12 (1.63) | 4 (50.00) |
| *Missing* | *0* | *0* |
| Previous familial breast lesion |  |  |
| None | 456 (62.04) | 4 (50.00) |
| Benign | 279 (37.96) | 4 (50.00) |
| *Missing* | *0* | *0* |

**Abbreviations**

MEDAS = Mediterranean Diet Adherence Screener

**Notes:**

Column percentages are reported for non-missing values; the proportion of missing data is calculated on the total sample size and reported separately.
